# Recombination-Mediated Telomere Maintenance in *Saccharomyces cerevisiae* Is Not Dependent on the Shu Complex

**DOI:** 10.1371/journal.pone.0151314

**Published:** 2016-03-14

**Authors:** Paula M. van Mourik, Jannie de Jong, Danielle Agpalo, Clémence Claussin, Rodney Rothstein, Michael Chang

**Affiliations:** 1 European Research Institute for the Biology of Ageing, University of Groningen, University Medical Center Groningen, Groningen, The Netherlands; 2 Department of Genetics and Development, Columbia University Medical Center, New York, United States of America; Tulane University Health Sciences Center, UNITED STATES

## Abstract

In cells lacking telomerase, telomeres shorten progressively during each cell division due to incomplete end-replication. When the telomeres become very short, cells enter a state that blocks cell division, termed senescence. A subset of these cells can overcome senescence and maintain their telomeres using telomerase-independent mechanisms. In *Saccharomyces cerevisiae*, these cells are called ‘survivors’ and are dependent on Rad52-dependent homologous recombination and Pol32-dependent break-induced replication. There are two main types of survivors: type I and type II. The type I survivors require Rad51 and maintain telomeres by amplification of subtelomeric elements, while the type II survivors are Rad51-independent, but require the MRX complex and Sgs1 to amplify the C_1–3_A/TG_1–3_ telomeric sequences. Rad52, Pol32, Rad51, and Sgs1 are also important to prevent accelerated senescence, indicating that recombination processes are important at telomeres even before the formation of survivors. The Shu complex, which consists of Shu1, Shu2, Psy3, and Csm2, promotes Rad51-dependent homologous recombination and has been suggested to be important for break-induced replication. It also promotes the formation of recombination intermediates that are processed by the Sgs1-Top3-Rmi1 complex, as mutations in the *SHU* genes can suppress various *sgs1*, *top3*, and *rmi1* mutant phenotypes. Given the importance of recombination processes during senescence and survivor formation, and the involvement of the Shu complex in many of the same processes during DNA repair, we hypothesized that the Shu complex may also have functions at telomeres. Surprisingly, we find that this is not the case: the Shu complex does not affect the rate of senescence, does not influence survivor formation, and deletion of *SHU1* does not suppress the rapid senescence and type II survivor formation defect of a telomerase-negative *sgs1* mutant. Altogether, our data suggest that the Shu complex is not important for recombination processes at telomeres.

## Introduction

Telomeres are nucleoprotein structures at the ends of linear chromosomes that help a cell distinguish a natural chromosome end from a DNA double-strand break (DSB) [1]. In *Saccharomyces cerevisiae*, the telomeric DNA consists of 300 ± 75 bp of C_1–3_A/TG_1–3_ repetitive sequences, with the G-rich strand extending to form a 3′ single-stranded overhang [2]. The subtelomeric regions also contain middle repetitive X and Y′ elements. An X element is found at all chromosome ends, while the Y′ elements are found in zero to four tandem copies between an X element and the terminal telomeric repeats [3]. Telomeres are maintained by a specialized reverse transcriptase called telomerase, whose core subunits are a catalytic protein component (Est2) and an RNA subunit (TLC1), which can extend telomeres by adding TG_1–3_ repeats to the 3′ overhang [4, 5]. In cells lacking telomerase, telomeres shorten progressively during each cell division due to incomplete end-replication and nucleolytic degradation [6]. When the telomeres become very short, cells enter a state that blocks cell division, termed senescence. A subset of these cells can overcome senescence and maintain their telomeres using recombination-based processes, becoming ‘survivors’ [7]. There are two main types of survivors: type I and type II. Both types require Rad52-dependent homologous recombination (HR). Type I survivors also require Rad51, Rad54, and Rad57, and maintain telomeres by amplification of subtelomeric Y′ elements [7, 8]. Formation of type II survivors, which exhibit amplification of the C_1–3_A/TG_1–3_ sequences, is Rad51-independent, but requires the MRX complex (Mre11, Rad50, and Xrs2), Rad59, and Sgs1 [8–11]. The type I subtelomeric and type II telomeric amplification patterns can be easily distinguished on a genomic blot probing for telomeric sequences. Both types of survivors also require the DNA polymerase δ subunit Pol32, which is required for break-induced replication (BIR) [12]. BIR can be Rad51-dependent or Rad51-independent, suggesting that type I and type II survivors maintain telomeres through Rad51-dependent BIR and Rad51-independent BIR, respectively [13, 14]. Telomerase-negative cells lacking Rad52, Rad51, Rad54, Rad57, Sgs1, or Pol32 also senesce very rapidly, indicating that these proteins are important at telomeres even before the emergence of survivors [7, 10, 11, 15, 16].

The Shu complex, which consists of Shu1, Shu2, Psy3, and Csm2, interacts indirectly with Rad51 through the Rad51 paralogues Rad55-Rad57 to stimulate Rad51 filament attachment to the single-stranded DNA, which is essential for the homology recognition and strand invasion steps of HR [17–19]. When any of these four genes are deleted, a higher rate of mutations and increased number of genome rearrangements are observed [20, 21]. The Shu complex also promotes the formation of recombination intermediates that are processed by the Sgs1-Top3-Rmi1 complex, as mutations in the *SHU* genes can suppress various *sgs1*, *top3*, and *rmi1* mutant phenotypes [21, 22].

Given the role of the Shu complex in recombination-mediated processes, and the role of recombination proteins in senescence and survivor formation [23], we hypothesized that the Shu complex also functions during senescence and survivor formation. Surprisingly, we find that the Shu complex affects neither the rate of senescence nor survivor formation significantly. Furthermore, the deletion of *SHU1* does not suppress the rapid senescence and type II survivor formation defect of a telomerase-negative *sgs1Δ* mutant. Taken together, our findings suggest that the Shu complex does not normally function in recombination-mediated processes at telomeres.

## Materials and Methods

### Yeast strains and growth conditions

Standard yeast media and growth conditions were used [24, 25]. Strains used in this study are listed in Table 1 and all are *RAD5* derivatives of W303 (*ade2-1 can1-100 his3-11*,*15 leu2-3*,*112 trp1-1 ura3-1 RAD5*) [26, 27].

**Table 1:** Yeast strains used in this study.

Strain name	Relevant genotype
MCY574	*MAT***a**/α *est2ΔURA3/EST2 shu1ΔHIS3/SHU1*
MCY575	*MAT***a**/α *tlc1ΔHIS3/TLC1 shu2ΔURA3/SHU2*
MCY576	*MAT***a**/α *tlc1ΔHIS3/TLC1 psy3ΔkanMX/PSY3*
MCY577	*MAT***a**/α *tlc1ΔHIS3/TLC1 csm2ΔkanMX/CSM2*
YPM1	*MAT***a**/α *est2ΔURA3/EST2 rad51ΔkanMX/RAD51 shu1ΔHIS3/SHU1*
YPM2	*MAT***a**/α *tlc1ΔHIS3/TLC1 rad51ΔkanMX/RAD51 shu2ΔURA3/SHU2*
YPM3	*MAT***a**/α *est2ΔURA3/EST2 rad59ΔkanMX/RAD59 shu1ΔHIS3/SHU1*
YPM4	*MAT***a**/α *tlc1ΔHIS3/TLC1 rad59ΔkanMX/RAD59 shu2ΔURA3/SHU2*
YPM5	*MAT***a**/α *est2ΔURA3/EST2 sgs1ΔnatMX/SGS1 shu1ΔHIS3/SHU1*

### Liquid culture senescence assay

Senescence assays in liquid culture were performed essentially as previously described [28, 29]. Each senescence assay started with *est2Δ/EST2* or *tlc1Δ/TLC1* heterozygous diploids that were propagated for at least 50 generations before sporulation to ensure that telomeres were at a stable equilibrium length. Freshly dissected spores were allowed to form colonies on YPD agar plates after 2 days of growth at 30°C. Cells from these colonies were serially passaged in liquid YPD medium at 24-h intervals. For each passage, the cell density of each culture was measured by optical density (calibrated by cell counting using a haemocytometer) or by using a CASY Cell Counter, and the cultures were diluted back into fresh YPD medium at a cell density of 2 x 10^5^ cells/ml. Senescence was plotted with respect to population doublings (PDs). PD was used as a metric rather than time (e.g. days in culture) because senescence caused by telomere shortening is related to cell division, not time. In addition, the use of PDs prevents mutations that only alter the rate of cell division from being mistakenly interpreted as having an effect on the rate of senescence.

### Generation of survivors on agar plates

Diploids were propagated and sporulated as in the liquid culture senescence assays. Cells from freshly dissected spores were streaked on YPD plates and grown at 30°C for 3 days. Individual colonies were restreaked for 5–6 times to allow for survivor generation.

### Telomere PCR and telomere length measurements

Yeast genomic DNA was isolated using a Wizard Genomic DNA Purification Kit (Promega). Y′ telomeres and telomere VI-R were amplified by PCR as previously described [30, 31]. Telomere PCR products were separated by agarose gel electrophoresis and average telomere length was determined as previously described [32].

### Telomere genomic blot

Genomic DNA was isolated, digested with *Xho*I, separated on a 1% (w/v) agarose gel, and transferred to a Hybond-N^+^ membrane (GE Healthcare). The membrane was then hybridized to a telomere-specific (5′-CACCACACCCACACACCACACCCACA-3′) digoxigenin-labeled probe.

## Results and Discussion

### The Shu complex does not affect senescence or survivor formation

**Fig 1:**
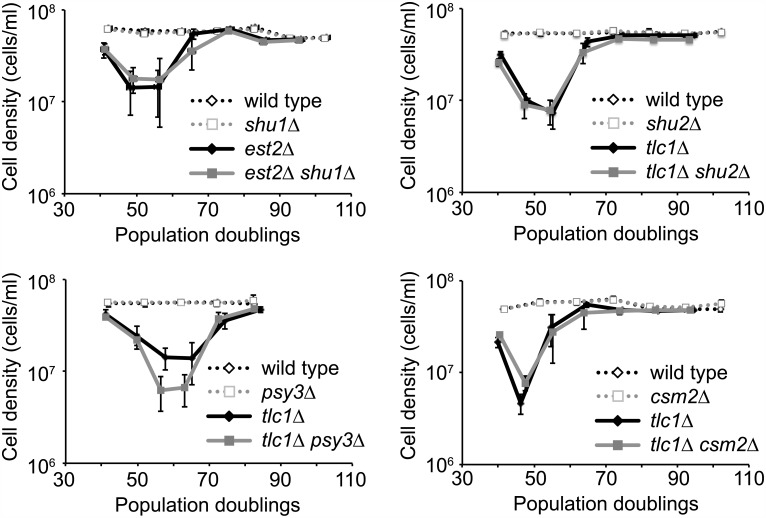
The Shu complex does not influence the rate of senescence or survivor formation. *est2Δ/EST2 shu1Δ/SHU1* (top left), *tlc1Δ/TLC1 shu2Δ/SHU2* (top right), *tlc1Δ/TLC1 psy3Δ/PSY3* (bottom left), and *tlc1Δ/TLC1 csm2Δ/CSM2* (bottom right) diploid strains were sporulated to generate the indicated haploid strains, which were subjected to a liquid culture senescence assay as described in the Materials and Methods. For each experiment, 2–3 isolates of each telomerase-positive strain and 4–5 isolates of each telomerase-negative strain were followed. The mean cell densities and standard errors of the means are shown.

To investigate whether the Shu complex plays a role during the process of senescence and in the formation of survivors in telomerase-negative cells, we first performed liquid culture senescence assays. Diploid strains that are deleted for one copy of either *EST2* or *TLC1* and also one copy of one of the four *SHU* genes were sporulated and the haploid progeny were propagated in liquid culture for several days (see Materials and Methods). In each case, the rate of senescence and survivor formation of *est2Δ* or *tlc1Δ* mutants was not affected by deletion of any of the *SHU* genes (Fig 1). Since all four *shu* mutants behaved similarly, subsequent experiments were performed with only one or two *shu* mutants.

**Fig 2:**
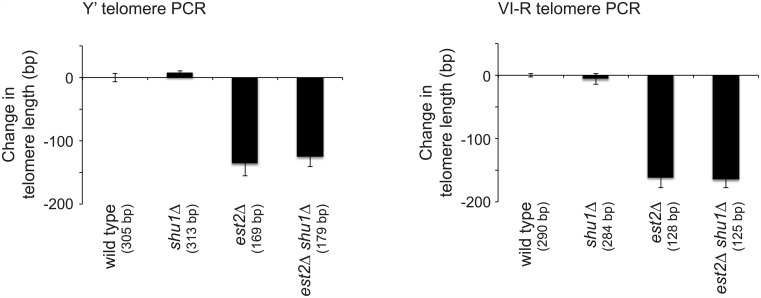
Deletion of *SHU1* does not affect telomere length in the presence or absence of telomerase. Strains of the indicated genotypes, generated from the sporulation of an *est2Δ*/*EST2 shu1Δ*/*SHU1* diploid, were assayed for telomere length by Y′ and VI-R telomere PCR after being passaged for approximately 35 generations. The change in telomere length, compared to wild-type telomere length, was quantified and plotted. Mean ± standard error for 3–4 independent isolates for each genotype are shown. Raw mean telomere length values are given in parentheses.

We next determined whether the Shu complex influences telomere length homeostasis or telomere shortening in the absence of telomerase. We measured the telomere length of wild type, *shu1Δ*, *est2Δ*, and *est2Δ shu1Δ* haploid strains approximately 35 generations after the sporulation of an *est2Δ/EST2 shu1Δ/SHU1* diploid. Deletion of *SHU1* did not affect either telomere length homeostasis of telomerase-positive cells or the telomere shortening of *est2Δ* cells (Fig 2).

Although our liquid culture senescence assays revealed that telomerase-negative *shu* mutants could form survivors (Fig 1), we wished to determine whether both types of survivors could be formed. We constructed *est2Δ rad51Δ shu1Δ*, *tlc1Δ rad51Δ shu2Δ*, *est2Δ rad59Δ shu1Δ*, and *tlc1Δ rad59Δ shu2Δ* strains and passaged them several times on solid medium. As mentioned above, Rad51 is required for the growth of type I survivors [8], so we can test whether deletion of *SHU1* or *SHU2* prevents type II survivor formation in a *rad51Δ* background. Likewise, since Rad59 is required for the growth of type II survivors [8], we can test whether deletion of *SHU1* or *SHU2* prevents type I survivor formation in a *rad59Δ* background. All mutants were able to recover from senescence and form survivors, indicating that neither type I nor type II survivors depend on the Shu complex for their formation.

To further validate that the Shu complex does not affect type I or type II survivor formation, we analyzed by genomic blot the telomeres of *est2Δ* and *est2Δ shu1Δ* survivors generated by serial passaging on solid medium after the sporulation of an *est2Δ/EST2 shu1Δ/SHU1* diploid strain. 71 *est2Δ* single mutants and 69 *est2Δ shu1Δ* double mutants were followed. Both *est2Δ* and *est2Δ shu1Δ* survivors were able to form type I and type II survivors, and for both genotypes, type I survivors were more abundant (Table 2), as previously reported [9, 33]. We did observe a small increase in type II survivor formation in the absence of *SHU1*, but this effect is not statistically significant (*X*^2^ = 1.49, *P* = 0.11). Thus, we conclude that the Shu complex does not play a major role in type I or type II survivor formation.

**Table 2:** Type II survivor frequencies in *est2Δ* and *est2Δ shu1Δ* cells.

Genotype	Type II frequency
***est2Δ***	5.6% (4/71)
***est2Δ shu1Δ***	13.0% (9/69)

### Deletion of *SHU1* does not rescue the rapid senescence and type II survivor formation defect in *est2Δ sgs1Δ* cells

**Fig 3:**
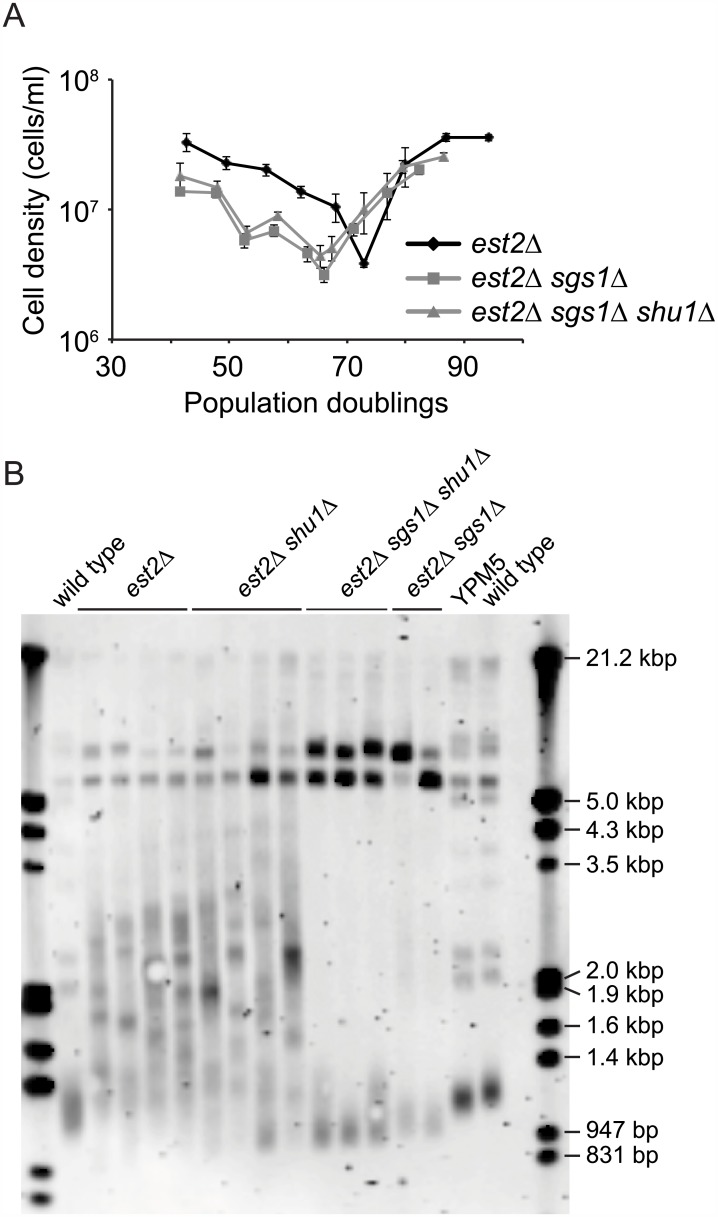
Rapid senescence and type II survivor formation defect of *est2Δ sgs1Δ* cells are not rescued by deletion of *SHU1*. (*A*) Strains for the indicated genotypes, generated from the sporulation of an *est2Δ/EST2 sgs1Δ/SGS1 shu1Δ/SHU1* (YPM5) diploid, were subjected to a liquid culture senescence assay. (*B*) A telomere genomic blot was performed on genomic DNA from strains of the indicated genotypes. The *est2Δ*, *est2Δ shu1Δ*, *est2Δ sgs1Δ shu1Δ*, *est2Δ sgs1Δ* strains were first passaged for 8 days in a liquid culture senescence assay to generate survivors. A haploid wild-type strain is included (on both sides of the blot), along with the YPM5 diploid.

Telomerase-negative cells lacking Sgs1 senesce rapidly and fail to form type II survivors [10, 11]. Since mutations in *SHU* genes can rescue various aspects of the *sgs1* mutant phenotype [21], we investigated whether the rapid senescence and type II survivor formation defect of telomerase-negative *sgs1Δ* mutants could be rescued by the deletion of *SHU1*. An *est2Δ/EST2 sgs1Δ/SGS1 shu1Δ/SHU1* diploid was sporulated to generate haploid meiotic progeny that were followed in a liquid culture senescence assay. The *est2Δ sgs1Δ* and *est2Δ sgs1Δ shu1Δ* mutants senesce at the same rate, and faster than an *est2Δ* single mutant (Fig 3A). The telomeres of the survivors were also analyzed by genomic blotting (Fig 3B). Type I survivors exhibit short telomeres and strong hybridization at 5.2 kb and 6.7 kb, which is due to amplification of the tandemly repeated Y′ short and Y′ long elements, respectively. The telomeres of type II survivor are extended and very heterogeneous in size. Since type II survivors grow much better than type I survivors, they outcompete the type I survivors in a liquid culture senescence assay [9, 33]. Thus, all *est2Δ* and *est2Δ shu1Δ* survivors generated this way are type II. The *est2Δ sgs1Δ* strains formed only type I survivors, as expected because deletion of *SGS1* prevents type II survivor formation [10, 11]. Deletion of *SHU1* did not rescue the inability of *est2Δ sgs1Δ* mutants to form type II survivors. Taken together, these results indicate that the Shu complex does not function upstream of Sgs1 with regards to senescence and survivor formation.

Overall, our findings indicate that the Shu complex does not play an important role during senescence and survivor formation. This result is surprising given the role of recombination proteins in these processes. In particular, the Shu complex is known to promote Rad51 filament formation [17–19], and Rad51 is needed to prevent rapid senescence and for type I survivor formation [8, 15], but telomerase-negative *shu* mutants do not show a similar phenotype (Fig 1 and Table 2). However, *shu* mutants are much less sensitive to DNA damaging agents than *rad51Δ* and *rad52Δ* mutants. In addition, spontaneous Rad51 focus formation is only down twofold in a *shu1Δ* strain [34], and while the Shu complex stimulates the loading of Rad51 onto RPA-coated single-stranded DNA *in vitro*, it is not absolutely required [19]. Thus, in the absence of the Shu complex, suboptimal Rad51 filament formation may be sufficient to delay senescence and promote survivor formation in telomerase-null cells. Nevertheless, it has recently been observed that the deletion of *PSY3* partially suppresses telomere elongation in *cdc9-1* mutants [35], indicating that the Shu complex may have a role at telomeres in certain situations.

Our work raises intriguing questions about what substrates the Shu complex acts on. It has been suggested that the Shu complex functions in BIR [35, 36]. If so, it would be interesting to determine why it does not apparently affect BIR-mediated survivor formation. Of course, cells may regulate BIR differently at telomeres than at DSBs. Alternatively, telomeres resemble one-ended DSBs, and the Shu complex may only function when both ends of a DSB are present. If this is the case, it will be interesting to figure out how the Shu complex differentiates between one-ended and two-ended DSBs. Finally, while the role of recombination in telomerase-independent telomere maintenance is clear, it is much less obvious why recombination proteins are needed to prevent accelerated senescence. The discovery that the Shu complex is not important during senescence implies that only some recombination activities are important, which adds another piece to solving this puzzle.

## Supporting Information

S1 Dataset Raw data for Fig 1. (XLSX)

S2 Dataset Raw data for Fig 2. (XLSX)

S3 Dataset Raw data for Fig 3A. (XLSX)
